# Palmitate drives mitochondrial and ER stress through disruption of the CD73-Adenosine axis

**DOI:** 10.21203/rs.3.rs-9902042/v1

**Published:** 2026-06-15

**Authors:** Shantiece Dawson, Sonia Batan, Nina Adams, Sergei Bombin, Erivan S. Ramos-Junior, Ana Carolina Morandini

**Affiliations:** Augusta University; Augusta University; Augusta University; Augusta University; Augusta University; Augusta University

**Keywords:** palmitate, adenosine, mitochondria, endoplasmic reticulum stress, CD73, inflammation

## Abstract

**Background:**

Metabolic disorders associated with elevated saturated fatty acids are linked to chronic inflammatory diseases, including periodontitis, yet the mechanisms connecting lipotoxic stress to gingival inflammation remain unclear. This study investigated how palmitate-induced metabolic stress affects purinergic signaling, mitochondrial function, and endoplasmic reticulum (ER) stress in murine gingival fibroblasts (mGF), and whether adenosine modulates these effects.

**Methods:**

mGF were treated with BSA control, palmitate, IL-1β, or palmitate plus IL-1β, followed by bulk RNA sequencing, Seahorse metabolic analysis, biochemical assays, and transmission electron microscopy.

**Results:**

Palmitate suppressed expression of key adenosine-generating ectoenzymes and purinergic signaling genes, including Cd73 (Nt5e), Cd39 (Entpd1), Adk, Ada, and adenosine receptors. Concurrently, palmitate amplified IL-1β-induced inflammatory mediators such as Cxcl1, Cxcl2, Cxcl5, Ccl2, and Il6. Gene ontology analysis demonstrated enrichment of pathways related to innate immune activation, oxidative stress, mitochondrial dysfunction, ER stress, and purine metabolism. Palmitate also induced intracellular lipid accumulation and mitochondrial dysfunction, evidenced by reduced NAD+/NADH ratio, increased mitochondrial reactive oxygen species (ROS), elevated protein oxidation, and increased proton leak despite enhanced electron transport chain protein expression. Ultrastructural analyses revealed swollen mitochondria, ER expansion, and increased ER–mitochondrial associations. Mechanistically, palmitate activated the Perk–eIF2α–Atf4 ER stress pathway, increasing phosphorylation of Perk and eIF2α and elevating Atf4 expression. Extracellular adenosine attenuated mitochondrial ROS accumulation, reversed Perk and Atf4 activation, improved mitochondrial respiration, and preserved ER and mitochondrial ultrastructure.

**Conclusions:**

Palmitate disrupts the Cd73-adenosine axis while promoting mitochondrial dysfunction, oxidative stress, and Perk-mediated ER stress in gingival fibroblasts. Adenosine signaling protects against lipotoxic-induced ER stress, highlighting the Cd73-adenosine pathway as a potential therapeutic target in metabolically driven periodontal inflammation.

## Introduction

The role of metabolic dysfunction in chronic inflammatory illnesses, such as obesity-related periodontal disease, is increasingly recognized [1, 2]. Under conditions of metabolic stress, elevated circulating free fatty acids (FFAs), especially saturated fatty acids like palmitate build up and cause oxidative stress, lipotoxicity, and cellular dysfunction [3, 4]. A common saturated FFA in Western diets, palmitate is frequently employed in experimental models to cause metabolic stress and mitochondrial dysfunction in a variety of cell types [5, 6]. Excessive intake of palmitate increases reactive oxygen species (ROS) generation, modifies mitochondrial function, disrupts lipid homeostasis, and activates stress signaling pathways. Together, these consequences deteriorate tissue health [7, 8]. The precise effect of lipotoxic stress on gingival stromal cells is yet unknown despite the fact that obesity and metabolic syndrome are associated with increased inflammation and a higher risk of tissue damage in periodontal tissues [9, 10].

Palmitate and other saturated FFAs not only affect metabolism but also significantly elevate inflammatory signals. Previous studies have shown that palmitate increases levels of pro-inflammatory cytokines and chemokines by triggering oxidative stress, and inflammasome signaling[11]. High fatty acid levels and persistent low-grade inflammation frequently coexist in obesity-related disorders, which may also enhance inflammation of periodontal tissues [12]. Extracellular purinergic signaling has been identified as a crucial endogenous mechanism that promotes tissue adaptability to stress and restricts excessive inflammation. Through the activation of adenosine receptors such A2a and A2b receptors, adenosine produced by CD39/CD73-mediated ATP hydrolysis has anti-inflammatory and tissue-protective effects [13–16].

Adenosine production mediated by the ectoenzyme CD73 is also becoming more closely associated with cellular stress tolerance, resilience and mitochondrial homeostasis[17]. The ectoenzyme CD73 and adenosine receptor signaling control oxidative stress reactions, redox equilibrium, and mitochondrial respiration [18, 19]. Previous research by our group showed that by reducing oxidative stress and maintaining mitochondrial function, CD73-derived adenosine signaling helps to maintain mitochondrial integrity and protects against inflammatory tissue damage[20]. Additionally, higher cellular survival during inflammatory stress, decreased ROS generation, and improved mitochondrial bioenergetics have all been linked to adenosine receptor activation [14, 21]. Disruption of the CD73-adenosine pathway during metabolic stress may have a significant impact on mitochondrial adaptability and inflammatory consequences in gingival fibroblasts since mitochondria are key regulators of energy production, redox signaling, and inflammatory responses.

Mitochondrial dysfunction and endoplasmic reticulum (ER) stress are very closely related, especially through signaling at mitochondria-associated ER membranes (MAMs) [22–24]. Damage to the mitochondria caused by oxidative stress and excessive metabolic activity disrupts calcium homeostasis, protein folding, and redox status, thereby triggering the unfolded protein response (UPR) [25]. The PERK-eIF2α-ATF4 pathway is a main UPR route that helps cells adapt to oxidative and metabolic stress [26, 27]. In chronic disease models, ongoing PERK activation leads to an accumulation of ROS, triggers inflammation, and disrupts metabolism [28, 29]. Earlier studies found saturated fatty acids, such as palmitate, induce ER stress and activate the PERK pathway in metabolic tissues [5, 6]. Still, it is unclear whether the same pathways mediate lipotoxic stress in gingival fibroblasts.

In this study, we screened for differentially expressed genes in response to the free fatty acid palmitate in the presence or absence of the classical pro-inflammatory cytokine IL-1β, which is relevant to chronic inflammatory diseases and periodontal pathogenesis. We explored the mechanisms underlying palmitate-induced metabolic dysregulation in murine gingival fibroblasts. This study identified purinergic-metabolic targets that were significantly altered following palmitate exposure and found that mitochondrial dysfunction and ER stress were central components of the cellular response. Overall, our findings provide new insights into how lipotoxicity may contribute to periodontal cell dysfunction through coordinated disruption of mitochondrial and ER homeostasis. Further, here we identify the CD73–adenosine pathway as an important regulator of metabolic and inflammatory stress response in gingival fibroblasts.

## Material and Methods

### Cell Culture and drug treatments

Primary murine gingival fibroblasts (mGF) were obtained from murine gingival explants as previously described [20]. For this study, mGF obtained from the gingiva surrounding the maxillary second molar of 3–6 month-old C57Bl6 mice were isolated and cultured in Dulbecco’s Modified Eagle’s Medium (DMEM) supplemented with 20% fetal bovine serum and antibiotics. The mGF were cultured in a 5% CO_2_ incubator at 37°C and the growth media was replaced every other day. Experiments were performed using cells between the 2nd and 8th passages, at around 80% confluence and viable cells were automatically counted using a cell counter and Trypan Blue staining. Cells were seeded in uniformity of cell distribution the day before each experiment. For cell treatments the following reagents were used: 200 μM BSA Control for BSA Fatty Acid Complexes (5 mM) as a vehicle, 200 μM BSA-Palmitate Saturated Fatty Acid Complex (5 mM) (Cayman Chemical Company Inc, MI, USA, #29556 and #29558); 1 ng/mL mouse recombinant Il-1β (401-ML, R&D systems), 100μM Adenosine (A4036–5G, MilliporeSigma) combined with 10μM EHNA (E114–25MG, MilliporeSigma) to prevent adenosine degradation in our in vitro model as previously established in our lab [22]. Adenosine treatment was performed 5 min before the addition of Vehicle or Palmitate in the presence or absence of mouse recombinant Il-1β for the times indicated in each figure legend.

### RNA isolation and Bulk RNA-sequencing

Total RNA was extracted from samples using Invitrogen PureLink RNA Mini Kit (ThermoFisher Scientific) isolation method according to the manufacturer’s instructions. RNA concentration and purity were assessed using a NanoDrop spectrophotometer, and RNA integrity was evaluated using the Agilent 2100 Bioanalyzer. Only samples with RNA integrity number (RIN) ≥ 7 were used for downstream processing.

Full-length cDNA was generated using the SMART-Seq (Switching Mechanism at 5’ End of RNA Template) technology (Takara Bio) following the manufacturer’s protocol. Briefly, reverse transcription was performed using an oligo(dT) primer to selectively target polyadenylated mRNA. During first-strand synthesis, template-switching activity of the reverse transcriptase enabled the addition of a defined sequence at the 5’ end, allowing for full-length cDNA amplification. The resulting cDNA was amplified by PCR to generate sufficient material for library preparation. Sequencing libraries were prepared from amplified cDNA using a protocol guideline, amplified with indexed primers and purified. Final libraries were quantified and assessed for size distribution using Qubit fluorometric quantification and Agilent Tape Station. Sequencing was performed on an Illumina platform NovaSeq 6000 to generate paired-end reads (2 × 150 bp).

### Bioinformatics Methods:

Raw sequencing reads in BCL format were demultiplexed and converted to FASTQ files using Illumina’s bcl2fastq2 v2.20. Adapter sequences and low-quality bases were removed using Trimmomatic v0.39. The resulting high-quality reads were aligned to the GENCODE Mus musculus reference genome (GRCm39 vM33) using the STAR v2.7.10a aligner. A gene-level count matrix was then generated from the aligned reads using HTSeq v2.0.9. Differential gene expression analysis between experimental groups was conducted in R using the DESeq2 v1.40.2 package. To identify enriched biological pathways, Gene Set Enrichment Analysis (GSEA) was performed with the GSEA function from the clusterProfiler v4.8.1 package, using the Gene Ontology Biological Process reference gene set collection. Data visualization, including heatmaps, volcano plots, and further clustering analyses, was performed using ggplot2 and heatmap in R. Principal component analysis (PCA) was used to assess sample clustering and variability.

### Quantitative real-time PCR

RNA was extracted from 1×10^5^ cells/well with the Invitrogen PureLink RNA Mini Kit (ThermoFisher Scientific) according to the manufacturer’s instruction. Briefly, samples were lysed in equal volumes of lysis buffer and stored in a −80°C freezer for a few hours. Then, samples were homogenized by vortexing and centrifuging for 5 minutes at 4°C at 12,000xg. After adding 70% ethanol, RNA was extracted and purified using a fast spin-column workflow and kit-provided wash buffers. All samples were eluted in 30 μl of RNase-Free water stored in a −80°C freezer. Reverse transcription was performed using a SuperScript IV VILO Master Mix (ThermoFisher Scientific) to obtain cDNA from Nanodrop read samples. The quantitative PCR was performed using the following inventoried Taqman assays: mouse Nt5e: Mm00501910_m; mouse Cxcl1: Mm04207460_m1; mouse Ccl2: Mm00441242_m1, mouse Il6: Mm00446190_m; and mouse Gapdh: Mm99999915_g1 in a 10uL final volume using TaqMan Fast Advanced Master Mix in a StepOne Plus Real-Time PCR system (Applied Biosystems). Relative quantitation of the Gapdh reference gene versus the target gene was performed in duplex reactions and calculated using the comparative Ct (ΔΔCt) values to generate the RQ for each sample based on the established cycle threshold for each target. Analysis was performed using StepOne Plus software and Graph Pad Prism.

### Western blotting

#### Western blotting

For immunoblot analyses, 60uL of cold RIPA buffer (ThermoFisher) was used to obtain the total protein extract from 1 × 10^5^ cells/mL. Westerns blots were performed with an equal amount of the total protein (10 μg of total protein/lane for all *in vitro* samples) with Stain-Free all molecular weight gel (#4568124, Bio-Rad) using the Miniprotean system with Fast Blot settings in Mixed MW transfer (#1704150, Bio-Rad). Densitometry analysis was performed using ImageLab Software v.6.1 (Bio-Rad, Hercules, CA, USA) and normalized for the total protein, as indicated in each figure legend. The following antibodies with their respective dilutions were used for immunoblot experiments: Primary antibodies: Cd73 (1:2000, # PA5–85958; Invitrogen, Thermo Fisher Scientific, Waltham, MA, USA); Primary (Abcam, Cambridge, MA, USA): Anti-Adenosine Receptor A2a antibody [EP6182(2)] (1:1000, ab169756); Total OXPHOS Rodent Cocktail (1:1000, ab110413); Anti-xCT [EPR27115–64] (1:1000, ab307601); Primary (Cell Signaling, Beverly, MA, USA): Phospho-Perk (Thr980)(16F8) (1:1000, #3179), Perk (C33E10) (1:1000, #3192); Atf-4 (D4B8) (1:1000, #11815); Secondary antibodies (Cell Signaling): Anti-mouse IgG (HRP-linked) (1:1000, #7076), Anti-rabbit IgG (HRP) (1:3000, #7074). The OxyBlot protein oxidation detection kit (#S7150; Millipore Sigma) was used for immunodetection of protein oxidation through carbonyl groups, which is a hallmark of the oxidation status of proteins.

### Adenosine quantification

Adenosine levels were measured using the Adenosine Assay Kit (ab211094, Abcam) according to the manufacturer’s instructions. Following cell treatment with BSA or Palmitate with or without Il-1β, cells were washed once with 1X PBS and lysed in 300 μL of Adenosine Assay Buffer. Cells were collected and centrifuged at 12,000 × g for 10 minutes at 4°C, and the supernatant was collected. Supernatant samples were diluted as needed to fall within the assay range (samples were diluted 2x-50x in PBS1x). Then, 50 μL of standard or samples were added to a 96-well white flat-bottom plate, followed by the addition of 50 μL of Reaction Mix prepared according to the manufacturer’s instructions. The plate was incubated for 15 minutes at room temperature protected from light. Fluorescence intensity was measured using a Synergy H1 plate reader (BioTek) at excitation/emission wavelengths of 535/587 nm. Adenosine concentrations were calculated based on the standard curve provided with the kit and expressed in pmoL/uL.

### Oil Red O staining

Palmitate uptake was assessed by staining intracellular neutral lipids using Oil Red O Staining. Cells were stained with an Oil Red O Stain Kit (#KTORO, StatLab, USA) according to the manufacturer’s instructions. Briefly, cells were fixed in 10% neutral buffered formalin for 15 minutes at room temperature and washed three times with distilled water. Cells were then pretreated with 85% propylene glycol for 2 minutes, followed by rinsing with distilled water. Subsequently, cells were incubated with Oil Red O staining solution at 60°C for 5 minutes. After staining, slides were differentiated in 85% propylene glycol for 1 minute and rinsed with distilled water. Finally, slides were mounted using an aqueous mounting medium.

### Nad+ assay

For the NAD/NADH-Glo Assay (G9071; Promega Corporation, Madison, WI, USA), the mGFs were seeded in a white 96 well plate clear bottom at a cell density of 3×10^4^ cells/well. After cell treatments, a standard curve was generated using purified NAD^+^, prepared at a starting concentration of 2 mM in PBS and serially diluted as required. Treatment media were then removed and replaced with 50 μL of PBS per well. Then, 50 μL of NAD/NADH-Glo Detection Reagent was added to each well, and the plate was gently mixed to ensure complete cell lysis. The plate was incubated at room temperature for 15–30 minutes. Luminescence was measured using Synergy H1 plate reader (BioTek).

### CellRox and Mitotracker fluorescence staining

After washing the mGF with 1x PBS, they were placed in MitoTracker^®^ Red CMXRos (300nM) and CellROX (5μM) working solution (ThermoFisher) and incubated in the dark at 37°C for 30 min. Following incubation, the cells were washed again with PBS. Subsequently, they were fixed with 4% paraformaldehyde (PFA) at room temperature for 15 min, washed three times with 1x PBS, and then mounted on glass slides with DAPI-mounting media (Vector). Images were acquired using a confocal laser scanning microscope (Leica).

### Transmission Electron Microscopy

Transmission electron microscopy of the mGF was performed for cells exposed to cell treatments with either BSA as a vehicle of Palmitate with and without Il-1β and with or without adenosine according to previously described [17] and as detailed in the figure legends.

### Seahorse assay

Mitochondrial function of mGF after cell treatments with either 200μM BSA Control as a vehicle or 200μM BSA-Palmitate Saturated Fatty Acid Complex with or without 100μM Adenosine combined with 10μM EHNA was performed using a Seahorse XFe96 Extracellular Flux Analyzer (Seahorse Bioscience) as previously described (Paladines et al., 2023). Briefly, 4 × 10^4^ cells were seeded into the Seahorse XF Cell Culture Microplate (Agilent Technology) in OPTiMEM, one day before the experiment. Cell treatments were performed as described in drug treatments section. For analysis, cells were resuspended in XF assay media (Agilent Technology) supplemented with 10 mM glucose (Sigma-Aldrich), 1 mM pyruvate (Sigma-Aldrich), and 2 mM glutamine (Sigma-Aldrich). The Cell Mito Stress Test was performed using 1.5μM oligomycin, 1.0μM FCCP (carbonyl cyanide-p-trifluoromethoxy-phenyl-hydrazone), 0.5μM rotenone, and 0.5μM antimycin A (RotAA) from Agilent Technologies. All results were normalized per the total protein in each well after the assay using the BCA method (Pierce Protein Biology). Mitochondrial stress was measured via Oxygen Consumption Rate (OCR) and Extracellular Acidification Rate (ECAR), respectively, in pmol/min/μg of protein. Metabolic parameters were exported and calculated according to the manufacturer’s instructions (Agilent Technologies) using the Seahorse Wave desktop software (Agilent Technologies).

### Data analysis

Statistical analysis was performed using the GraphPad Prism v10 software (GraphPad, San Diego, CA, USA) using ANOVA followed by multiple comparison tests. Data are presented as mean ± S.D. The cell number per well was chosen based on cell density optimization experiments for the specific assay. The significance level of p is indicated in each graph and their respective figure legends (*p < 0.05; **p < 0.01; ***p < 0.001;****p < 0.0001).

## Results

### Palmitate dampens adenosine-generating ectoenzymes while promoting inflammatory and metabolic stress markers in murine gingival fibroblasts (mGF)

**Figure 1:**
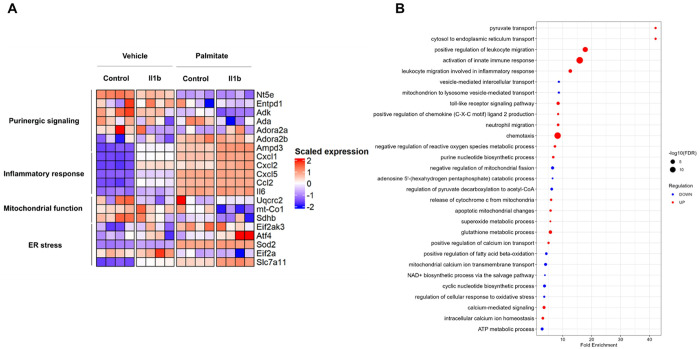
Palmitate-induced metabolic dysregulation decreases CD73 and increases inflammatory chemokines and mitochondrial stress genes in murine gingival fibroblasts (mGF). (A) Heatmap of bulk RNAseq analysis showing the 20 most significant genes regulated in mGFs stimulated with or without palmitate (200μM) in the presence or absence of 1ng/mL IL-1β for 24h. Blue shades demonstrate downregulation of Nt5e (Cd73) in palmitate-stimulated mGF regardless of IL-1β challenge. Red color corresponds to upregulated mRNA expression for most genes related to inflammatory response and mitochondrial metabolism. (B) Gene ontology analysis of activate and suppressed pathways comparing Control mGF and Palmitate-stimulated mGF.

To simulate metabolic dysfunction, mGF were treated with BSA-conjugated palmitate, a well-established inducer of metabolic stress [6], followed by bulk RNA sequencing to characterize transcriptional changes associated with this dysfunctional metabolic state. In parallel, cells were treated with BSA (control), and with or without IL-1β, in order to assess the combined effects of metabolic and inflammatory stress. Principal component analysis (PCA) revealed a clear separation of samples based on treatment conditions (Fig. S1A). Replicates within each group clustered tightly, demonstrating good reproducibility across samples. We observed genes linked to purinergic signaling, inflammation, mitochondrial function, and cell stress to be significant different between palmitate-stimulated versus control samples (Fig. 1A and Fig S1B). The expression of Nt5e (Cd73), the enzyme that hydrolyzes adenosine monophosphate into extracellular adenosine, dropped significantly after palmitate treatment. This decrease continued when IL-1β was added, showing that Cd73-mediated purinergic signaling is impaired during metabolic and inflammatory stress. Other purinergic pathway components, such as Entpd1(Cd39), Adenosine kinase (Adk), Adenosine deaminase (Ada), and adenosine receptor genes Adora2a, and Adora2b, were also downregulated, indicating broader suppression of adenosine signaling pathway.

Moving to inflammatory response, IL-1β stimulation greatly increased pro-inflammatory genes such as Cxcl1, Cxcl2, Cxcl5, Ccl2, and Il6 compared to controls. Palmitate alone caused a minor increase, whereas with the addition of IL-1β, levels either remained the same or increased. This shows palmitate boosts IL-1β–driven inflammation (Fig. 1A). In addition to these changes, mitochondrial gene expression also changed, with lower levels of genes related to complexes from the electron transport chain Uqcrc2, mt-Co1, and Sdhb in cells treated with IL-1β, especially when combined with palmitate. This suggests impaired mitochondrial function and energy production. Furthermore, genes related to the integrated stress response and Endoplasmic reticulum (ER) stress - Eif2ak3, Atf4, Eif2a, and Slc7a11- were upregulated, especially under combined treatment. Overall, palmitate significantly reduced Cd73-dependent purinergic signaling while causing inflammation and metabolic stress, suggested by the enhancement of genes related to mitochondrial damage and ER stress.

Gene Ontology (GO) enrichment analysis was performed on differentially expressed genes to identify the biological processes associated with the observed gene expression changes (Fig. 1B and Fig S1B). Pathway analysis highlighted multiple inflammatory and immune-related processes, including innate immune activation, neutrophil and leukocyte migration, and chemotaxis (Fig. 1B). Enrichment in Toll-like receptor signaling and chemokine pathways were also observed which fits with the inflammatory gene expression changes in Fig. 1A and Fig S1B. Along with these, several metabolic and mitochondrial pathways were altered, including pyruvate transport, regulation of pyruvate decarboxylation to Acetyl-CoA, and mitochondrial dynamics along with apoptosis processes such as cytochrome c release, indicating disrupted mitochondrial homeostasis. Pathways related to redox balance and oxidative stress, including superoxide and glutathione metabolism, were enriched, indicating increased oxidative stress under these conditions (Fig. 1B).

Importantly, processes related to purine nucleotide metabolism and adenosine pathways, such as purine nucleotide biosynthetic process and adenosine catabolic process, were also significantly affected, supporting the identified suppression of Cd73-mediated purinergic signaling in Fig. 1A and Fig S1B. Enrichment of pathways involved in ER transport, calcium-mediated signaling, and ion homeostasis suggests activation of ER stress and integrated stress responses. Together, these data demonstrate that inflammatory and metabolic stress are concomitant and correlated with coordinated alterations in immune activation, mitochondrial function, oxidative stress, and purinergic signaling.

#### Validation of transcriptomic findings reveals dysregulation of the CD73-adenosine-A2aR axis

**Figure 2:**
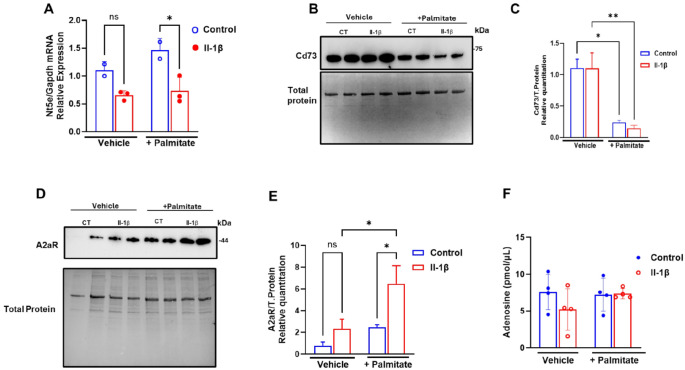
Palmitate stimulation decreases ectonucleotidase Cd73 and increases adenosine A2a receptor by IL-1β-stimulated mGF. (A) The ectonucleotidases Cd73 mRNA expression after 6h and (B) immunoblot in mGF challenged with palmitate (200μM) or BSA (vehicle) with or without 1ng/mL of Il-1β for 24h. (C) Densitometry analysis show relative Cd73 quantitation compared to total protein. (D) Immunoblot and (E) respective densitometric analysis of adenosine receptor A2a in mGF challenged with palmitate (200μM) or BSA (vehicle) with or without 1ng/mL of Il-1β for 24h. (F) Adenosine levels in mGF challenged with palmitate (200μM) or BSA (vehicle) with or without 1ng/mL of Il-1β for 24h. Data are presented as mean ± S.D. (*p <0.05; **p <0.01).

To validate RNA-seq results (Fig. 1), we assessed key components of the purinergic pathway in all experimental groups. Palmitate alone did not noticeably change Cd73 mRNA levels, while the decrease seen with IL-1β was more significant when palmitate + IL-1β was added (Fig. 2A). In contrast, Cd73 protein levels were significantly reduced following palmitate exposure, as shown by immunoblot analysis (Fig. 2B) and respective densitometric quantification (Fig. 2C). The adenosine A2aR protein expression was increased under these conditions. Immunoblot analysis showed higher A2aR levels following palmitate and IL-1β treatment (Fig. 2D), as reflected in densitometric analysis (Fig. 2E), suggesting a possible compensatory increase in adenosine receptor signaling, possibly due to low Cd73 levels. To assess the functional consequence of these changes, extracellular adenosine levels were measured. Although Cd73 and A2aR expression were altered, extracellular adenosine levels showed no significant difference following palmitate or IL-1β treatment (Fig. 2F). Together, these results validate the transcriptomic findings and highlight a shift in purinergic signaling, marked by reduced Cd73 expression in the presence of palmitate-induced metabolic stress and increased A2aR levels in mGF.

### Palmitate leads to lipid accumulation in mGF and enhances the IL-1β-induced inflammatory response.

**Figure 3:**
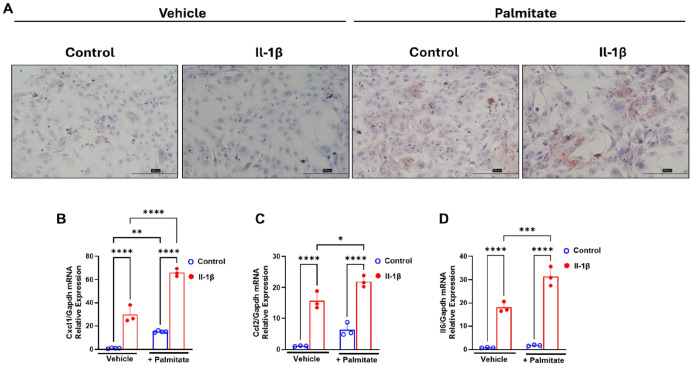
Palmitate stimulation leads to fat accumulation (lipid droplets content) intracellularly in mGF and increases IL-1β-induced inflammatory cytokines such as CxCl1, Ccl2 and Il-6. (A) Oil Red O Staining demonstrate detected lipid content stained in red inside palmitate-stimulated mGF with and without Il-1β (1ng/mL). Relative quantitative mRNA levels of (B)Cxcl1 (C) Ccl2 and (D) Il-6 in mGF stimulated with Vehicle (BSA) or Palmitate (200μM) in the presence or absence of Il-1β (1ng/mL) for 6h. Data are presented as mean ± S.D. (*p <0.05; **p <0.01; ***p<0.001; ****p<0.0001).

To assess the lipid uptake by mGF, Oil Red O staining was used to evaluate lipid accumulation in mGF treated with BSA (control), palmitate, IL-1β, or IL-1β + palmitate. As expected, IL-1β treatment alone did not induce remarkable lipid accumulation while palmitate treatment resulted in a marked increase in intracellular lipid accumulation, as evidenced by enhanced Oil Red O staining (Fig. 3A). The co-treatment with IL-1β and palmitate showed a similar increase in lipid deposition as observed with palmitate alone, confirming that palmitate effectively induces a lipotoxic state in mGF. Corroborating Fig. 1, we confirmed bulk RNAseq data for the inflammatory response showing that IL-1β stimulation led to the upregulation of pro-inflammatory genes, including Cxcl1 (Fig. 3B), Cxcl2 (Fig. 3C), and Il6 (Fig. 3D) which was even stronger and more significant with the addition of palmitate. In other words, palmitate increased the expression of IL-1 β-induced inflammatory genes, suggesting that metabolic and inflammatory stress act together as suggested in our bulk RNA-seq data.

#### Palmitate induces mitochondrial stress regardless of IL-1β treatment in mGF

**Figure 4:**
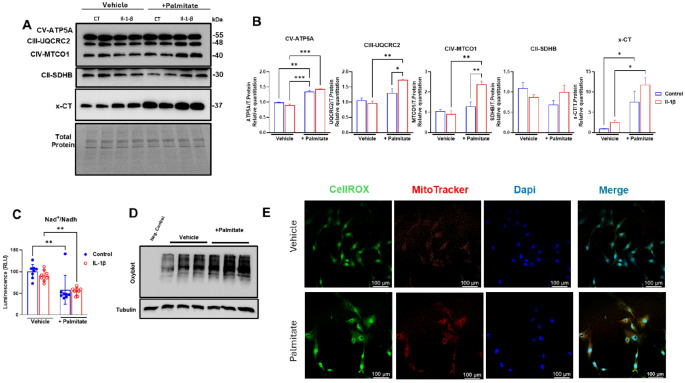
Palmitate leads to mitochondrial stress in Il-1β-stimulated cells. **(A)** Mitochondrial electron transport chain (ETC) complexes expression in mGF stimulated with Vehicle (BSA) or Palmitate (200μM) in the presence or absence of Il-1β(1ng/mL) for 24h. (B) Densitometry analysis of mitochondrial ETC complex subunits shown in Figure 4A. **(C)** Nad^+^/Nadh ratio of mGF stimulated with Vehicle (BSA) or Palmitate (200μM) in the presence or absence of Il-1β(1ng/mL) for 24h. **(D)** Immunoblot detection of oxidative reactions through Oxyblot Protein Oxidation kit comparing mGF extracts with Vehicle (BSA) or Palmitate (200μM) for 6h. **(E)** Immunofluorescence staining for total ROS through CellROX (green) and mitochondria via Mitotracker staining (red) for mGF stimulated with Vehicle (BSA) or Palmitate (200μM) for 6h. Nuclei of mGF counterstained with Dapi (blue). Data are presented as mean ± S.D. (*p <0.05; **p <0.01; ***p<0.001).

To evaluate mitochondrial function, we looked at ETC protein levels in mGF under BSA (control), palmitate, IL-1β, or both. Protein levels of multiple ETC complexes were enhanced following palmitate treatment, and this increase was more evident in the presence of IL-1β (Fig. 4A, B), indicating more expression of mitochondrial complexes in the presence of palmitate. Because the expression of the ETC complexes alone is not sufficient to infer about function, we next measured the NAD^+^/NADH ratio as a readout of cellular redox state. Palmitate treatment led to a reduction in the NAD^+^/NADH ratio, both in IL-1β-treated and untreated cells (Fig. 4C), suggesting accumulation of oxidative stress. To determine whether oxidative stress was increased, protein oxidation was assessed using an OxyBlot assay. We observed increased protein oxidation in palmitate-treated cells compared to control (Fig. 4D). CellROX staining also showed higher ROS levels under the same conditions (Fig. 4E). Co-staining with MitoTracker showed that this increase in ROS was associated with mitochondria, supporting the presence of mitochondrial oxidative stress (Fig. 4E).

### Palmitate alters ER and mitochondrial structure and activates PERK-ATF4 signaling.

**Figure 5:**
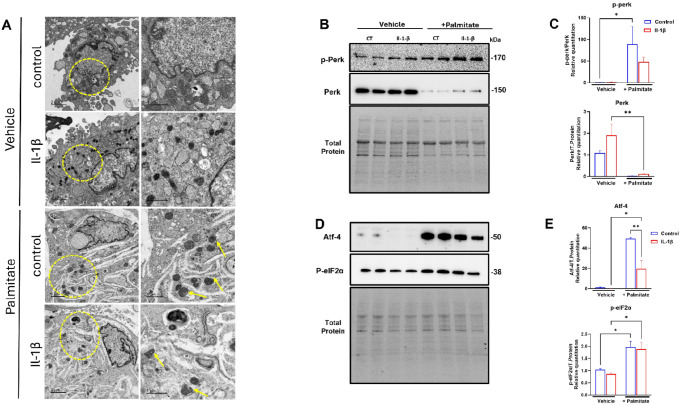
Palmitate affects endoplasmic reticulum and mitochondrial structure by inducing expansion of intracisternal space of ER and leading to swollen mitochondria via Perk/Atf-4 pathway. **(A)** Electron microscopy images from Vehicle or Palmitate-stimulated mGF with or without Il-1β(1ng/mL) for 24h. Left panel images show cells in low magnification and right panel images show high magnification closeup areas indicated by the dashed yellow circle. Yellow arrows point to areas of ER-mitochondrial tethering with evidence of swollen stressed mitochondria. **(B)**Immunoblot of p-Perk and total Perk in mGF challenged with palmitate (200μM) or BSA (vehicle) with or without 1ng/mL of Il-1β for 24h with the respective **(C)** densitometry analysis of p-Perk relative to total Perk and Total Perk relative to total Protein as a loading control. **(D)**Immunoblot of Atf-4 and p-eiF2α in mGF challenged with palmitate (200μM) or BSA (vehicle) with or without 1ng/mL of Il-1β for 24h with the respective **(E)** densitometry analysis relative to Total protein levels. Data are presented as mean ± S.D. (*p <0.05; **p <0.01).

We observed increased levels of ETC complex proteins, which may reflect changes in mitochondrial structure or function. To better understand this, we next examined ultrastructural changes by electron microscopy. In palmitate and IL-1β + palmitate-treated cells, the ER appeared expanded, with increased intracisternal space compared with control (Fig. 5A). Mitochondria in these cells also showed a swollen, stressed morphology. Higher magnification images highlighted regions where ER and mitochondria were closely associated, suggesting altered ER mitochondrial interactions which is a visual sign of ER stress.

To examine ER stress signaling, we measured PERK activation. Palmitate treatment increased Perk phosphorylation compared to control, and this effect was maintained in the presence of IL-1β (Fig. 5B, C). We next looked at Atf4 and pEiF2 α protein levels, which are downstream markers of the Perk pathway. ATF4 and phosphorylated eIF2α levels were higher with palmitate treatment (Fig. 5D, E), consistent with activation of the Perk-mediated ER stress response. Together these results suggest that palmitate induces ER and mitochondrial ultrastructural changes via Perk-Atf4 ER stress signaling pathway.

#### Adenosine attenuates palmitate-induced mitochondrial dysfunction and ER stress in mGF

**Figure 6:**
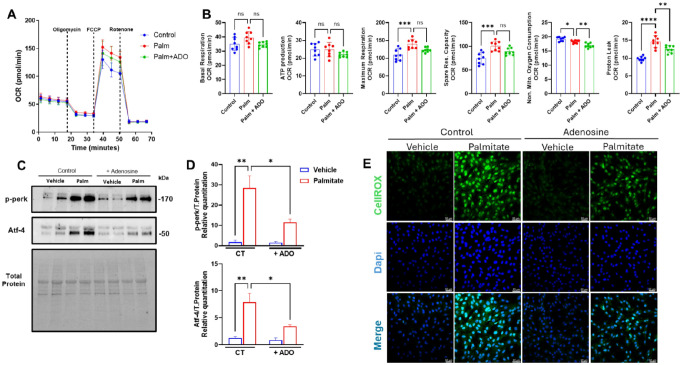
Adenosine reduces the increased proton leak induced by palmitate in mGF and dampens markers of ER stress p-Perk and Atf-4. **(A)** Seahorse analysis of Oxygen Consumption Rate (OCR) of mGF during Mito Stress Test in the presence of absence of Palmitate (200μM) with or without 100μM adenosine (ADO) for 6h. (B) Representative graphs of Mito Stress test parameters between control, Palmitate (Palm) or Palmitate+Adenosine (Palm+ADO). **(C)** Immunoblot for p-Perk and Atf-4 of mGF in the presence or absence of Palmitate (200μM) with or without 100μM adenosine (ADO) for 24h. Densitometry analysis of **(D)** p-Perk and Atf-4 relative to the total protein as a loading control. Data are presented as mean ± S.D. (*p <0.05; **p <0.01). **(E)** Immunofluorescence staining for total ROS through CellROX (green) for mGF stimulated with Vehicle (BSA) or Palmitate (200μM) for 6h. Nuclei of mGF counterstained with Dapi (blue).

We further evaluated mitochondrial function by real-time metabolic seahorse analysis. Palmitate affected maximal mitochondrial respiration, increasing proton leak in mGF. The observed increase in oxygen consumption associated with proton leak in palmitate-treated cells suggests reduced mitochondrial efficiency (Fig. 6A, B).

Given the link between mitochondrial dysfunction and ER stress, we next examined the effect of adenosine in activation of the PerK pathway. Palmitate-induced levels of phosphorylated Perk and Atf 4 were reduced in the presence of adenosine (Fig. 6C–D), suggesting a decrease in ER stress signaling. Further supporting evidence for protective role of adenosine against palmitate-induced cellular stress was observed by CellROX staining (Fig. 6E). Intracellular ROS levels were higher in palmitate-treated mGF. This increase was reduced in the presence of adenosine, with lower fluorescence intensity compared to palmitate alone (Fig. 6E).

### Adenosine preserves ER-mitochondrial structure and reduces palmitate-induced ultrastructural changes in mGF.

**Figure 7:**
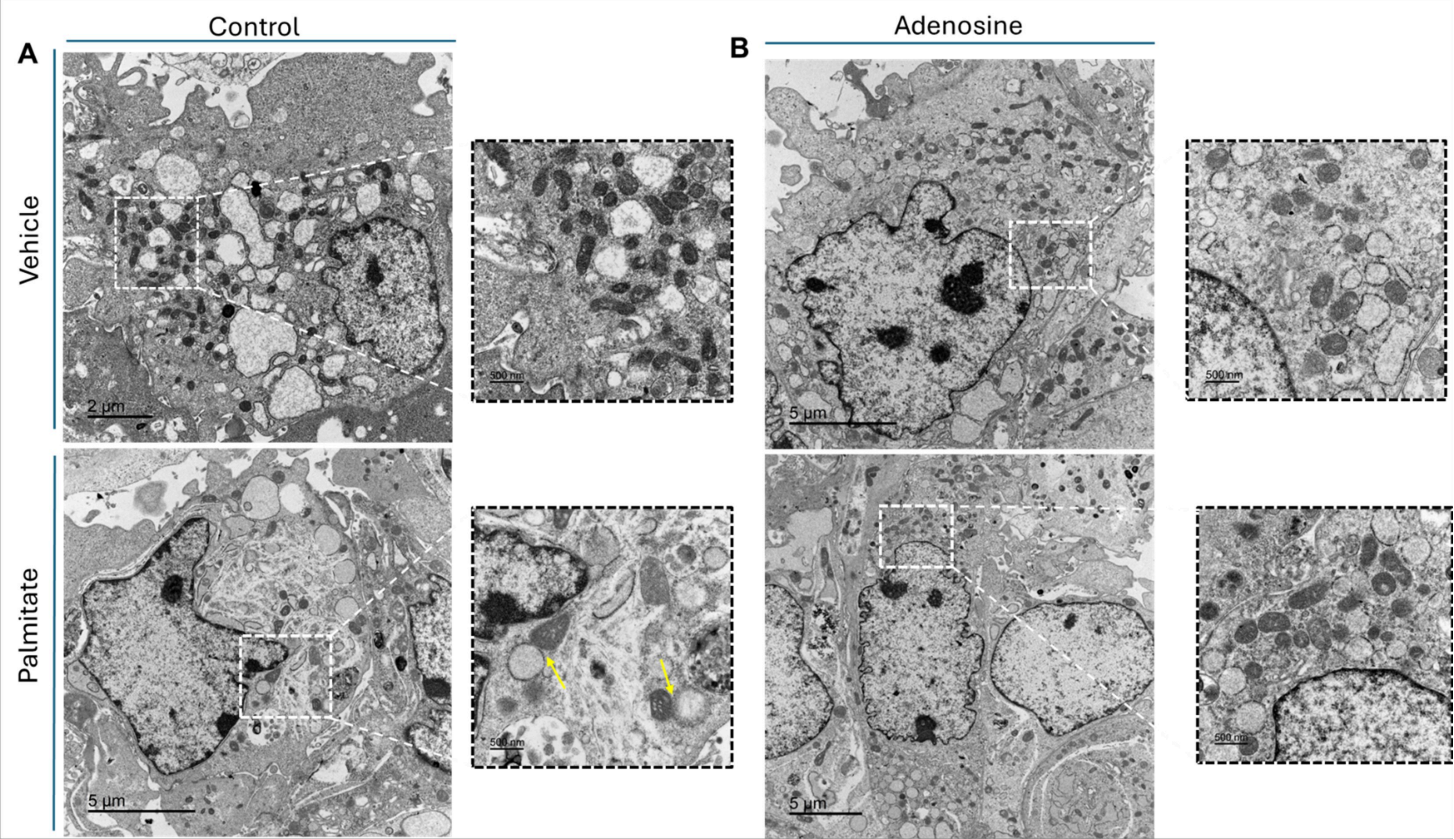
Adenosine prevents palmitate-induced signs of metabolic stress through reversing ER-mitochondria tethering and preservation of ER-mitochondrial structure. Transmission electron microscopy images of mGF in the presence or absence of **(A)** Vehicle (BSA) or Palmitate (200μM) **(B)** with or without 100μM adenosine (ADO) for 6h. Corresponding high magnification white dashed areas are shown in detail. Yellow arrows indicate regions of ER-mitochondrial tethering.

Transmission electron microscopy images showed changes in cell structure after palmitate treatment. Compared to vehicle, palmitate-treated mGF had disrupted ER and mitochondrial morphology, with more areas where the ER and mitochondria appeared closely associated (Fig. 5). With adenosine, the overall structure looked more preserved, with clearer ER and mitochondrial organization and fewer regions of close association (Fig. 7A, B). At higher magnification, palmitate-treated cells showed swollen mitochondria and expanded ER compartments, whereas these features were not present with adenosine treatment.

## Discussion

In this study, we demonstrate that palmitate-induced metabolic stress profoundly alters purinergic signaling, mitochondrial homeostasis, and ER stress responses in gingival fibroblasts, while simultaneously amplifying IL-1β-driven inflammatory signaling. Mechanistically, our data support a model in which lipotoxic stress suppresses the Cd73–adenosine axis, promotes oxidative and mitochondrial dysfunction, activates the Perk–eIF2α–Atf4 pathway of the unfolded protein response (UPR), and enhances inflammatory responsiveness. Importantly, extracellular adenosine restored mitochondrial and ER integrity, indicating that extracellular purinergic signaling functions as a protective pathway against lipotoxic injury and metabolic stress in gingival fibroblasts.

A central finding of this study is the suppression of Cd73-dependent purinergic signaling under palmitate exposure. The ectoenzyme Cd73 (Nt5e) is a critical ectonucleotidase that converts extracellular AMP into adenosine, thereby regulating anti-inflammatory and tissue-protective signaling through adenosine receptors, particularly A2aR and A2bR adenosine receptors. Reduced Cd73 expression following palmitate and IL-1β exposure suggests that metabolic stress impairs the ability of gingival fibroblasts to generate extracellular adenosine. This observation is consistent with previous studies showing that obesity-associated lipotoxicity and chronic inflammation dysregulate nucleotide metabolism[30, 31] and compromise adenosinergic immunoregulation[16]. Downregulation of additional purinergic genes, including Entpd1 (Cd39), Ada, Adk, Adora2a and Adora2b, further indicates broad disruption of extracellular nucleotide turnover and receptor responsiveness.

Interestingly, despite reduced Cd73 protein expression, extracellular adenosine concentrations were not significantly altered. This may reflect compensatory mechanisms that maintain extracellular adenosine homeostasis despite impaired enzymatic generation. For example, intracellular adenosine release through nucleoside transporters or reduced adenosine catabolism may buffer extracellular levels transiently[32]. Alternatively, extracellular adenosine measurements may not fully capture the altered levels because of methodological limitations and due to the extremely short half-life of this nucleoside[33]. Notably, A2a receptor protein expression increased following palmitate and IL-1β treatment, potentially representing a compensatory response aimed at preserving anti-inflammatory signaling under conditions of reduced adenosine availability. Similar compensatory receptor upregulation has been reported during inflammatory and metabolic stress in other cell types such as T cells [15].

Palmitate also markedly potentiated IL-1β-induced inflammatory expression, including Cxcl1, Cxcl2, Cxcl5, Ccl2, and Il6. Saturated fatty acids such as palmitate are well-established activators of inflammatory pathways through mechanisms involving Toll-like receptor 4, NF-κB activation, ROS generation, and inflammasome signaling [34, 35]. The enrichment of Toll-like receptor signaling and leukocyte chemotaxis pathways observed in GO analysis strongly supports this mechanism. Importantly, our findings suggest that metabolic stress enhances gingival fibroblasts hyper-inflammatory profile rather than acting solely as an independent inflammatory trigger. This synergistic interaction between lipotoxicity and cytokine signaling may be particularly relevant in obesity-associated periodontal inflammation, where elevated circulating fatty acids coexist with chronic inflammatory mediators [12, 36].

Mitochondrial dysfunction emerged as a major consequence of palmitate exposure[37]. In this study, transcriptomic analysis revealed altered expression of genes associated with the electron transport chain (ETC), mitochondrial dynamics, pyruvate metabolism, and oxidative stress pathways. Although ETC protein levels increased following palmitate treatment, Seahorse analysis demonstrated increased proton leak and impaired mitochondrial efficiency, indicating that elevated ETC abundance does not necessarily translate into improved oxidative phosphorylation capacity[38]. This apparent discrepancy may reflect increased ETC protein expression due to early lipotoxic stress which can be often accompanied by inefficient electron transport and enhanced electron leakage [8]. The reduction in the NAD+/NADH ratio together with increased protein oxidation and mitochondrial ROS strongly supports the presence of mitochondrial oxidative stress[39]. The observed increase in mitochondrial ROS by CellROX/MitoTracker co-localization further suggests that mitochondria are a major source of oxidative stress in palmitate-treated mGF. Elevated ROS can damage mitochondrial DNA, proteins, and membrane lipids, further perpetuating mitochondrial dysfunction and inflammatory signaling[40]. Moreover, oxidative stress is closely linked to activation of ER stress pathways[41], particularly the PERK branch of the UPR[42].

Indeed, our ultrastructural and biochemical analyses demonstrated robust activation of ER stress signaling in response to palmitate. Transmission electron microscopy revealed ER dilation and swollen mitochondria, classical morphological features associated with unresolved ER stress and mitochondrial injury [8, 43]. Increased ER–mitochondrial proximity observed in palmitate-treated cells may indicate altered mitochondria-associated membrane (MAM) dynamics [24]. MAMs serve as critical signaling platforms coordinating calcium transfer, lipid metabolism, mitochondrial dynamics, and apoptotic signaling between the ER and mitochondria [23]. Under pathological conditions, excessive ER-mitochondrial coupling can exacerbate calcium overload and mitochondrial ROS production[44], thereby amplifying cellular stress responses.

At the molecular level, palmitate activated the PERK–eIF2α–ATF4 pathway, as evidenced by increased PERK phosphorylation and elevated ATF4 and phospho-eIF2α levels. PERK activation is a hallmark adaptive response to ER stress that transiently attenuates protein translation while selectively promoting stress-responsive transcriptional programs through ATF4 [45]. Sustained activation of this pathway, however, contributes to oxidative stress, metabolic dysfunction, and inflammatory signaling. Upregulation of Slc7a11(xCT) in the transcriptomic analysis and protein validation is also notable, as this transporter is commonly induced downstream of ATF4 to support glutathione synthesis and redox adaptation[46]. The enrichment of glutathione metabolism and superoxide pathways further supports activation of integrated stress response mechanisms aimed at counteracting oxidative injury [27].

Importantly, extracellular adenosine exerted significant protective effects against palmitate-induced stress responses. Adenosine reduced PERK phosphorylation, decreased ATF4 activation, attenuated ROS accumulation, improved mitochondrial respiratory efficiency, and preserved ER and mitochondrial ultrastructure. These findings align with previous reports demonstrating that adenosine signaling can limit oxidative stress[47] and mitochondrial dysfunction[17] in addition to its well-known role in suppression of inflammatory signaling pathways [14, 19]. Activation of A2A receptors has also been shown to inhibit ER stress-mediated apoptosis and reduce ROS production in multiple cell types [14, 19]. The protective effects of adenosine on ER–mitochondrial architecture are particularly intriguing. Excessive ER–mitochondrial contacts have been implicated in metabolic diseases, including obesity, diabetes, and fatty liver disease, where they contribute to calcium dysregulation and mitochondrial injury [24]. Our ultrastructural observations suggest that adenosine may help normalize ER–mitochondrial communication during lipotoxic stress, thereby reducing propagation of stress signals between these organelles.

Given the limitations of an in vitro experimental setting, collectively, these findings support a mechanistic model in which palmitate-induced lipotoxicity disrupts mitochondrial metabolism and extracellular purinergic signaling, leading to oxidative stress, Perk-dependent ER stress activation, and heightened inflammatory responsiveness in gingival fibroblasts. Reduced Cd73 expression may diminish adenosine-mediated tissue protection, thereby exacerbating mitochondrial and ER dysfunction. Restoration of adenosine signaling attenuates these pathological responses, highlighting the Cd73-adenosine axis as a potential therapeutic target for metabolic inflammation in periodontal tissues. Ongoing experiments in our lab are focused on the in vivo validation in models of obesity-associated periodontal disease to confirm the translational relevance of these findings.

## Supplementary Material

Supplementary Files

This is a list of supplementary files associated with this preprint. Click to download.

- Supplementarypalmitatepaper.pdf

## Data Availability

The bulk RNA-seq dataset generated in this study is available through public repository NCBI Gene Expression Omnibus (GEO). The GEO ID is GSE334095.

## References

[1] Pihlstrom B. L., Michalowicz B. S., and Johnson N. W.. 2005. Periodontal diseases Lancet 366(9499):1809–1820. 10.1016/s0140-6736(05)67728-8 16298220

[2] Saito T., and Shimazaki Y.. 2007. Metabolic disorders related to obesity and periodontal disease.Periodontol 2000, 43, 254–266, 10.1111/j.1600-0757.2006.00186.x 17214843

[3] Listenberger L. L. 2003. Triglyceride accumulation protects against fatty acid-induced lipotoxicity. Proc Natl Acad Sci U S A 100(6):3077–3082. 10.1073/pnas.0630588100 12629214 PMC152249

[4] Ertunc M. E., and Hotamisligil G. S.. 2016. Lipid signaling and lipotoxicity in metaflammation: indications for metabolic disease pathogenesis and treatment. Journal Of Lipid Research 57(12):2099–2114. 10.1194/jlr.R066514 27330055 PMC5321214

[5] Cnop M., Foufelle F., and Velloso L. A.. 2012. Endoplasmic reticulum stress, obesity and diabetes. Trends In Molecular Medicine 18(1):59–68. 10.1016/j.molmed.2011.07.010 21889406

[6] Ly L. D. 2017. Oxidative stress and calcium dysregulation by palmitate in type 2 diabetes.Exp Mol Med, 49, (2), e291. 10.1038/emm.2016.157 28154371 PMC5336562

[7] Schönfeld P., and Wojtczak L.. 2008. Fatty acids as modulators of the cellular production of reactive oxygen species. Free Radical Biology And Medicine 45(3):231–241. 10.1016/j.freeradbiomed.2008.04.029 18482593

[8] Arruda A. P., Pers B. M., Parlakgül G., Güney E., Inouye K., and Hotamisligil G. S.. 2014. Chronic enrichment of hepatic endoplasmic reticulum-mitochondria contact leads to mitochondrial dysfunction in obesity. Nature Medicine 20(12):1427–1435. 10.1038/nm.3735 PMC4412031 25419710

[9] Suvan J., D’Aiuto F., Moles D. R., Petrie A., and Donos N.. 2011. Association between overweight/obesity and periodontitis in adults. A systematic review. Obesity Reviews 12(5):e381–404. 10.1111/j.1467-789X.2010.00808.x 21348914

[10] Keller A., Rohde J. F., Raymond K., and Heitmann B. L.. 2015. Association between periodontal disease and overweight and obesity: a systematic review. Journal Of Periodontology 86(6):766–776. 10.1902/jop.2015.140589 25672656

[11] Rogers L. M. 2020. Palmitate induces apoptotic cell death and inflammasome activation in human placental macrophages.Placenta, 90, 45–51, 10.1016/j.placenta.2019.12.009 32056551 PMC7034939

[12] Pamuk F., and Kantarci A.. 2022. Inflammation as a link between periodontal disease and obesity.Periodontol 2000, 90, (1), 186–196, 10.1111/prd.12457 35916870

[13] Antonioli L., Blandizzi C., Pacher P., and Haskó G.. 2013. Immunity, inflammation and cancer: a leading role for adenosine. Nature Reviews Cancer 13(12):842–857. 10.1038/nrc3613 24226193

[14] Haskó G., Linden J., Cronstein B., and Pacher P.. 2008. Adenosine receptors: therapeutic aspects for inflammatory and immune diseases. Nature Reviews. Drug Discovery 7(9):759–770. 10.1038/nrd2638 18758473 PMC2568887

[15] Ohta A., and Sitkovsky M.. 2014. Extracellular adenosine-mediated modulation of regulatory T cells.Front Immunol, 5, 304, 10.3389/fimmu.2014.00304 25071765 PMC4091046

[16] Antonioli L., Pacher P., Vizi E. S., and Hasko G.. 2013.CD39 and CD73 in immunity and inflammation. Trends Mol Med, 19, (6), 355–367, 10.1016/j.molmed.2013.03.005 23601906 PMC3674206

[17] Morandini A. C., Dawson S., Paladines N., Adams N., and Ramos-Junior E. S.. 2025. Adenosine A2a Receptor Stimulation Mitigates Periodontitis and Is Mitoprotective in Gingival Fibroblasts Promoting Cellular Resilience.Cells, 14, (16), 10.3390/cells14161266 PMC12384189 40862745

[18] Cekic C., and Linden J.. 2016. Purinergic regulation of the immune system. Nature Reviews Immunology 16(3):177–192. 10.1038/nri.2016.4 26922909

[19] Antonioli L., Pacher P., and Haskó G.. 2022. Adenosine and inflammation: it’s time to (re)solve the problem. Trends In Pharmacological Sciences 43(1):43–55. 10.1016/j.tips.2021.10.010 34776241

[20] Ramos-Junior E. S. 2023. The protective role of CD73 in periodontitis: preventing hyper-inflammatory fibroblasts and driving osteoclast energy metabolism. Front Oral Health 4:1308657. 10.3389/froh.2023.1308657 38152410 PMC10751373

[21] Eltzschig H. K., Bonney S. K., and Eckle T.. 2013. Attenuating myocardial ischemia by targeting A2B adenosine receptors. Trends In Molecular Medicine 19(6):345–354. 10.1016/j.molmed.2013.02.005 23540714 PMC3674126

[22] Paladines N. 2023. Metabolic reprogramming through mitochondrial biogenesis drives adenosine anti-inflammatory effects: new mechanism controlling gingival fibroblast hyper-inflammatory state. Frontiers In Immunology 14:1148216. 10.3389/fimmu.2023.1148216 37350964 PMC10282177

[23] Csordás G., Weaver D., and Hajnóczky G.. 2018. Endoplasmic Reticulum-Mitochondrial Contactology: Structure and Signaling Functions. Trends In Cell Biology 28(7):523–540. 10.1016/j.tcb.2018.02.009 29588129 PMC6005738

[24] Tubbs E., and Rieusset J.. 2017. Metabolic signaling functions of ER-mitochondria contact sites: role in metabolic diseases. Journal Of Molecular Endocrinology 58(2):R87–r106. 10.1530/jme-16-0189 27965371

[25] Hetz C. 2012. The unfolded protein response: controlling cell fate decisions under ER stress and beyond. Nature Reviews Molecular Cell Biology 13(2):89–102. 10.1038/nrm3270 22251901

[26] Harding H. P. 2003. An integrated stress response regulates amino acid metabolism and resistance to oxidative stress. Molecular Cell 11(3):619–633. 10.1016/s1097-2765(03)00105-9 12667446

[27] Pakos-Zebrucka K., Koryga I., Mnich K., Ljujic M., Samali A., and Gorman A. M.. 2016. The integrated stress response. Embo Reports 17(10):1374–1395. 10.15252/embr.201642195 27629041 PMC5048378

[28] Rozpedek W., Pytel D., Mucha B., Leszczynska H., Diehl J. A., and Majsterek I.. 2016. The Role of the PERK/eIF2α/ATF4/CHOP Signaling Pathway in Tumor Progression During Endoplasmic Reticulum Stress. Current Molecular Medicine 16(6):533–544. 10.2174/1566524016666160523143937 27211800 PMC5008685

[29] Urra H., Dufey E., Avril T., Chevet E., and Hetz C.. 2016. Endoplasmic Reticulum Stress and the Hallmarks of Cancer. Trends Cancer 2(5):252–262. 10.1016/j.trecan.2016.03.007 28741511

[30] Liu D. 2026. Nucleotide metabolic rewiring enables NLRP3 inflammasome hyperactivation in obesity.Science, 391, (6782), eadq9006, 10.1126/science.adq9006 41538457 PMC13034487

[31] Csoka B. 2014. A2B adenosine receptors prevent insulin resistance by inhibiting adipose tissue inflammation via maintaining alternative macrophage activation.Diabetes, 63, (3), 850–66. 10.2337/db13-0573 24194503 PMC3931402

[32] Lofgren L., Pehrsson S., Hagglund G., Tjellstrom H., and Nylander S.. 2018. Accurate measurement of endogenous adenosine in human blood. PLoS One 13(10):e0205707. 10.1371/journal.pone.0205707 30359421 PMC6201894

[33] Ramakers B. P. 2008. Measurement of the endogenous adenosine concentration in humans in vivo: methodological considerations. Current Drug Metabolism 9(8):679–685. 10.2174/138920008786049249 18855606

[34] Shi H., Kokoeva M. V., Inouye K., Tzameli I., Yin H., and Flier J. S.. 2006. .TLR4 links innate immunity and fatty acid-induced insulin resistance. J Clin Invest 116(11):3015–3025. 10.1172/jci28898 17053832 PMC1616196

[35] Wen H. 2011. Fatty acid-induced NLRP3-ASC inflammasome activation interferes with insulin signaling. Nature Immunology 12(5):408–415. 10.1038/ni.2022 21478880 PMC4090391

[36] Ramirez-Tortosa M. C. 2010. Periodontitis is associated with altered plasma fatty acids and cardiovascular risk markers. Nutrition, Metabolism, And Cardiovascular Diseases: Nmcd 20(2):133–139. 10.1016/j.numecd.2009.03.003 19500957

[37] Zhang X., Mao M., and Zuo Z.. 2022. Palmitate Induces Mitochondrial Energy Metabolism Disorder and Cellular Damage via the PPAR Signaling Pathway in Diabetic Cardiomyopathy. Diabetes Metab Syndr Obes 15:2287–2299. 10.2147/DMSO.S360931 35936050 PMC9355343

[38] Morandini A. C., and Ramos-Junior E. S.. 2024. Mitochondrial function in oral health and disease. Journal Of Immunological Methods 532:113729. 10.1016/j.jim.2024.113729 39067635 PMC12875047

[39] Yusri K., Jose S., Vermeulen K. S., Tan T. C. M., and Sorrentino V.. 2025. The role of NAD(+) metabolism and its modulation of mitochondria in aging and disease. NPJ Metab Health Dis 3(1):26. 10.1038/s44324-025-00067-0 40604314 PMC12177089

[40] Chen Y. S. 2025.ROS homeostasis in cell fate, pathophysiology, and therapeutic interventions. Mol Biomed, 6, (1), 89, 10.1186/s43556-025-00338-8 41162811 PMC12572517

[41] Santos C. X., Tanaka L. Y., Wosniak J., and Laurindo F. R.. 2009. Mechanisms and implications of reactive oxygen species generation during the unfolded protein response: roles of endoplasmic reticulum oxidoreductases, mitochondrial electron transport, and NADPH oxidase. Antioxidants & Redox Signaling 11(10):2409–2427. 10.1089/ars.2009.2625 19388824

[42] Saptarshi N., Porter L. F., and Paraoan L.. 2022.PERK/EIF2AK3 integrates endoplasmic reticulum stress-induced apoptosis, oxidative stress and autophagy responses in immortalised retinal pigment epithelial cells. Sci Rep, 12, (1), 13324, 10.1038/s41598-022-16909-6 35922637 PMC9349321

[43] Bravo R. 2011. Increased ER-mitochondrial coupling promotes mitochondrial respiration and bioenergetics during early phases of ER stress. Journal Of Cell Science 124(Pt 13):2143–2152. 10.1242/jcs.080762 21628424 PMC3113668

[44] Feissner R. F., Skalska J., Gaum W. E., and Sheu S. S.. 2009. Crosstalk signaling between mitochondrial Ca2 + and ROS. Front Biosci (Landmark Ed) 14(4):1197–1218. 10.2741/3303 19273125 PMC2683671

[45] Harding H. P., Zhang Y., and Ron D.. 1999. Protein translation and folding are coupled by an endoplasmic-reticulum-resident kinase.Nature, 397, (6716), 271–4. 10.1038/16729 9930704

[46] Ye P. 2014.Nrf2- and ATF4-dependent upregulation of xCT modulates the sensitivity of T24 bladder carcinoma cells to proteasome inhibition. Mol Cell Biol, 34, (18), 3421–3434, 10.1128/MCB.00221-14 25002527 PMC4135628

[47] Gholinejad M., Jafari Anarkooli I., Taromchi A., and Abdanipour A.. 2018. Adenosine decreases oxidative stress and protects H(2)O(2)-treated neural stem cells against apoptosis through decreasing Mst1 expression. Biomed Rep 8(5):439–446. 10.3892/br.2018.1083 29732147 PMC5921220

